# Falling Less in Kansas: Development of a Fall Risk Reduction Toolkit

**DOI:** 10.4061/2011/532079

**Published:** 2011-09-19

**Authors:** Teresa S. Radebaugh, Candace A. Bahner, Deborah Ballard-Reisch, Michael Epp, LaDonna S. Hale, Rich Hanley, Karen Kendrick, Michael E. Rogers, Nicole L. Rogers

**Affiliations:** ^1^Regional Institute on Aging, Wichita State University, 1845 Fairmount Street, Wichita, KS 67260, USA; ^2^Department of Physical Therapy, Wichita State University, 1845 Fairmount Street, Wichita, KS 67260, USA; ^3^Elliott School of Communication, Wichita State University, 1845 Fairmount Street, Wichita, KS 67260, USA; ^4^Envision Vision Rehabilitation Center, 610 N. Main Street, Wichita, KS 67203, USA; ^5^Department of Physician Assistant, Wichita State University, 1845 Fairmount Street, Wichita, KS 67260, USA; ^6^Harvey County Department on Aging, 800 N. Main Street, Newton, KS 67114, USA; ^7^Center for Physical Activity and Aging, Wichita State University, 1845 Fairmount Street, Wichita, KS 67260, USA; ^8^Aging Studies, Department of Public Health Sciences, Wichita State University, 1845 Fairmount Street, Wichita, KS 67260, USA

## Abstract

Falls are a serious health risk for older adults. But for those living in rural and frontier areas of the USA, the risks are higher because of limited access to health care providers and resources. This study employed a community-based participatory research approach to develop a fall prevention toolkit to be used by residents of rural and frontier areas without the assistance of health care providers. Qualitative data were gathered from both key informant interviews and focus groups with a broad range of participants. Data analysis revealed that to be effective and accepted, the toolkit should be not only evidence based but also practical, low-cost, self-explanatory, and usable without the assistance of a health care provider. Materials must be engaging, visually interesting, empowering, sensitive to reading level, and appropriate for low-vision users. These findings should be useful to other researchers developing education and awareness materials for older adults in rural areas.

## 1. Introduction

Falls by older adults are a significant public health issue. One-third of adults aged 65 and older fall each year [1]. For older adults, the most frequent cause of admission to a hospital for injury or trauma is a fall, and falls are the leading cause of injury-related death for adults in this age group [1]. Older adults located in rural and frontier areas of the US have increased risk because of limited access to health care providers and resources. Falling Less in Kansas used a three-step community-based participatory research approach to develop a fall awareness and risk reduction toolkit to be used by older residents of rural and frontier areas without the assistance of health care providers.

## 2. Background

 Falls can lead to a loss of independence, a decline in physical function and activity, higher rates of nursing home placement, and major economic consequences for individuals and families [2, 3]. The Centers for Disease Control and Prevention (CDC) report, “*The state of aging and health in America 2007*,” lists fall reduction as one of the top three areas that can significantly improve the quality of life for older adults [1].

**Figure 1:**
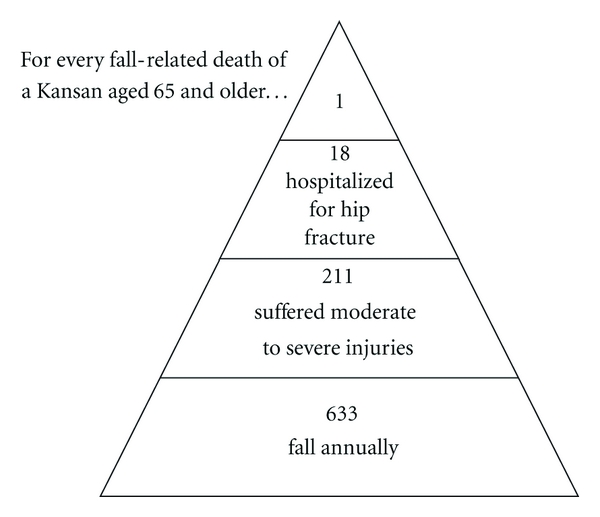
Falls and fall injuries of Kansas adults aged 65 and older, Kansas Health Statistics Report, February 2007. Figure 1 adapted from Figure 8, “*Falls and fall injuries among Michigan's older adults,*” Michigan Department of Community Health, October 2004.

In Kansas, fall trends are similar to national trends. Falls are the most common cause of trauma in Kansas and the second-leading cause of unintentional injury-related death, accounting for nearly 20% of such deaths. Of patients admitted to the hospital with a trauma-related fall, more than 40% were discharged to some type of institutional care. Between 2003 and 2007, the fall-related death rate for Kansans aged 85 or older was 158 deaths per 100,000 population [4]. Extrapolating from the Behavioral Risk Factor Surveillance System Kansas-specific survey, 57,000 adults aged 65 or older are estimated to have fallen during any three-month period. Further, of those who fell at least once, about 30% reported an injury that caused activity limitations for at least one day, or necessitated visiting a health care provider [5]. It is estimated that for every death from a fall-related injury in Kansas, 633 adults aged 65 or older fall (see Figure 1).

The CDC and National Council on Aging (NCOA) suggest evidence-based areas of focus for fall prevention programs, including (1) increasing physical activity, (2) evaluating and resolving medication issues, (3) identifying and referring vision problems, and (4) evaluating and resolving home safety issues [1, 6]. 

However, the strategies suggested by the CDC and others rely on trained healthcare professionals and resources that are scarce or nonexistent in rural areas. For example, the CDC's “*Preventing falls: How to develop community-based fall prevention programs for older adults*” states that program components should be delivered by physicians, optometrists, nurse practitioners, physician assistants, pharmacists, registered nurses, physical and occupational therapists, social workers, certified exercise instructors, and persons with exercise science/physical education degrees [7]. Also, these programs tend to be created from the “top down” by experts, with little input from potential users.

Programs tailored to urban areas will not reduce fall risk in rural areas which make up 80% of the US landmass and contain a population of approximately 70 million people [8]. Nearly two-thirds of the 105 Kansas counties are designated as rural or frontier and 85% are federally designated Health Professional Shortage Areas [9]. The range of healthcare services available to meet the needs of older adults in rural and frontier areas is narrower, less accessible, and more costly than in urban areas [10]. This healthcare disparity contributes to the lack of fall prevention programs in rural areas.

In almost three decades of fall research, there has been little attention to rural and frontier areas. A systematic review of the Cochrane Library, MEDLINE, and CINAHL databases through 2010 revealed only two articles discussing community-based fall prevention programs in a rural US setting [11, 12]. One low-cost, multifactorial program involved individualized and group education and materials regarding exercise, nutrition, and home safety, and the study intervention was designed to be implemented by a trained caregiver [12]. The other study utilized pharmacists and physicians to review electronic medical records (EMR) [11]. This was a randomized controlled study aimed at using EMR to identify at-risk patients and enable a medication-related intervention. 

The Falling LinKS Toolkit is a multicomponent toolkit that addresses the intervention areas recommended by CDC and NCOA and allows self-administration which is a distinctly different approach from these investigations. The objective of this initiative was to create a low-cost fall awareness and risk reduction toolkit that (a) integrates a community-based participatory approach with currently available, no-cost evidence-based strategies (i.e., First Step to Active Health and others discussed below), (b) is tailored to the resources and infrastructure of rural Kansas, and (c) meets the stated needs and preferences of older adults, their families, and community advocates. This initiative was branded as Falling Less in Kansas (Falling LinKS).

## 3. Methods

### 3.1. Research Partners and Teams

Falling LinKS is a research partnership among the Colleges of Education, Health Professions, Liberal Arts and Sciences, and the Regional Institute on Aging of Wichita State University, the Harvey County Kansas Department on Aging, and Envision Vision Rehabilitation Center.

Falling LinKS consists of two overlapping and multidisciplinary teams: the toolkit development team and the community-based participatory research (CBPR) team. The toolkit development team includes individuals with expertise in exercise science, pharmacy, physical and occupation therapy, low vision, public health, and gerontology. The CBPR team is headed by communication specialists with expertise in conducting community-grounded research.

The community partner is Harvey County Kansas, a rural county with a population of about 34,000 [13]. The race/ethnicity mix of the county population is somewhat less diverse than the state as a whole with about 85% White not Hispanic, 10% Hispanic or Latino, 2% Black, 0.7% American Indian or Alaska Native, and 0.7% Asian [13]. About 17% of the population is age 65 and older, compared to about 13% nationwide and in the state as a whole [13].

### 3.2. Community-Based Participatory Research Process

CBPR is an engaged research methodology involving community members in all phases of the research project: development, implementation, and evaluation [14]. 

A three-step iterative process was used to develop the Toolkit (see Table 1).

### 3.3. Data Collection and Analysis

The CBPR team gathered qualitative data regarding needs assessment and toolkit evaluation through key informant interviews and focus groups. Key informant interviews are individual interviews with knowledgeable people about the topic of interest. The interviews were conducted either in person or via telephone, were led by trained graduate students, and expanded upon, as needed, by the key informant through responses to open-ended questions. Interviews were audio recorded. The interviewer took handwritten or computer-based notes during the interview. Within 24 hours, a finalized data file was created by integrating the notes and audio recording. 

Focus groups are semistructured group interviews, guided by a facilitator, to address topics of interest. Roles and procedures were followed as outlined byClements-Nolle et al. [15]. A finalized data file was created through collaboration between the facilitator, recorder, and scribe where each reviewed the others' records and reconciled discrepancies [15].

Each CBPR team member conducted an inductive thematic analysis [16] to generate individual findings. Inductive analysis involves the team member immersing him/herself in the data and allowing themes to emerge without a priori assumptions. The objective is to understand the perspective of those interviewed and to identify commonalities and differences in their view points. Specifically, each CBPR team member conducted an independent, data-driven inductive analysis [16]. Then, the CBPR team members compared and contrasted their perceptions and worked collaboratively to integrate individual findings into the consensus summary reports that were provided to the toolkit development team.

#### 3.3.1. Step 1: Needs Assessment of Health Providers and Community Volunteers to Inform Initial Selection of Toolkit Materials

Step 1 consisted of 16 key informant interviews, one provider focus group (*n* = 11), and sustained communication with representatives from the Harvey County Department on Aging. The purpose was to determine local interest, need, and infrastructure/capacity, and to obtain data regarding preferences for toolkit development, dissemination, and implementation strategies. Potential key informants were identified and invited to participate by the Director of the Harvey County Department on Aging based on his knowledge of the residents and service providers in Harvey County. Interviews were held in February 2009 with state, county, and community senior service providers, county officials, healthcare providers, and aging specialists. The interviews were conducted according to a prespecified series of open ended questions addressing the Harvey County context, need/interest in a falls prevention program, infrastructure or county capacity to support a falls prevention program, preferred dissemination methods and collaborators. The interviews ranged in duration from 10 to 40 minutes. 

Based upon the data gathered, questions and protocols were finalized for one provider focus group, convened on June 16, 2009, to expand upon and verify findings from the key informant interviews. Focus group members (*N* = 11) were recruited from lists developed during the key informant interviews, but excluded previous interviewees. The focus group addressed the five content areas discussed during the key informant interviews and described above.

#### 3.3.2. Step 2: Needs Assessment of Older Adults to Further Inform Toolkit Development

The process for Step 2 was similar to Step 1. Step 2 consisted of 16 key informant interviews and one summative focus group (*n* = 19) to gain the perspectives of older adults on the same issues described in Step 1 plus their perspectives on falls, fall prevention, and naming strategies for the toolkit. Key informants were older adults identified as active, community leaders or identified as more isolated and “hard to reach.” Interviews occurred in July and August 2009, ranged from 10 to 69 minutes in duration (average = 29 minutes), and were conducted according to prespecified open ended questions.

The summative focus group was convened on August 17. Older adults were recruited from throughout the county; focus group participants were from throughout the county as well as participants in activities at a local senior center. The Chair of the Board of the Hesston Senior Center invited participants. Additionally, participants self selected in response to an invitation in the Hesston Senior Center newsletter. The focus group (*N* = 19 participants) lasted 43 minutes and addressed issues on naming strategies for the toolkit, perspectives on falls and on fall prevention programs.

#### 3.3.3. Step 3: Toolkit Assessment to Inform Refinement and the Final Toolkit

Toolkit assessment occurred through five formative focus groups and one summative focus group conducted in April 2010. Two formative focus groups were with healthcare providers and community volunteers similar to Step 1. Three were with older adults similar to Step 2. The summative focus group was conducted with service and health care providers. A total of 42 participants were included in Step 3.

## 4. Results

### 4.1. Step 1: Results from Health Providers and Community Volunteers

*The major recommendations of the Step  1 health providers and community volunteers were that the toolkit be practical, fun, attractive, easy to use, and address common sensory limitations*. Participants recommended that a successful program be positive, fun, and proactive; multilevel and à la carte to allow users and communities to select modules most appropriate for their circumstances and populations; offer practical solutions; engage users visually and kinesthetically; provide instructions for each tool and how it will directly benefit the individual user; address major misperceptions concerning falls (e.g., “falls happens to other people” or “falls are inevitable”); train community leaders to facilitate the toolkit; recognize diversity; and offer multilevel dissemination and recruitment strategies designed to reach diverse segments of the county population. Further, the toolkit materials should be clean, clear, and concise; engaging; not overly technical; offer practical instruction; respect common sensory limitations, especially related to low vision; not text heavy; and use multimedia specifically to enhance learning. Feedback indicated that the toolkit should be aimed at older adults, their families, and community advocates, and should be evidence based. 

Based upon the qualitative data gathered from Step 1, the toolkit development team selected, adapted, and organized existing peer-reviewed or federal agency instruments and procedures for fall education and awareness that would be appropriate for health care shortage rural and frontier areas. To address the four areas recommended by CDC and NCOA and to construct a multicomponent toolkit, the selected instruments included the First Step to Active Health (FSAH),Vial of Life and File of Life, the Functional Vision Screening Questionnaire, Amsler Grid, and CDC Check for Safety: A Home Fall Prevention Checklist for Older Adults (all discussed below).

### 4.2. Step 2: Results from Older Adults

*The major recommendations from the older adults in this Step focused on the actual construction of the toolkit.* Feedback indicated that the toolkit should be in color, spiral bound, and contain a pocket where additional information could be stored. Tools were packaged so they could be easily photocopied and still be readable. Based upon the qualitative data gathered in Step 2, the team continued to develop the toolkit, streamlined and refined toolkit instruments, and developed a draft with language at about a fifth-grade reading level.

### 4.3. Step 3: Results of Toolkit Assessment and Feedback

*The major recommendations from this Step addressed the visual usability of the toolkit, its “look,” and ensuring the toolkit was self-empowering.* The toolkit assessment focus groups offered recommendations related to visual enhancements and readability including: using expanded spacing, increasing font size in the medication checklists, condensing text to bullets and bullets to phrases wherever possible, consolidating information throughout the document, deleting the definition of a “fall” as it is self-evident and unnecessary, changing images as the people portrayed in the toolkit were “too old” and were not “attractive” enough, and incorporating more images within the content modules, except the physical activity module which already included many images.

Themes related to interest in the material and users' ability to relate to it included reduce emphasis on the “negative” consequences of falls, increase emphasis on empowering older adults to reduce risk and effect positive change, and provide specific action steps at the end of each module and a full action plan at the end of the toolkit. Further, the participants did not identify with the term “older adult.” Participants felt the toolkit was valuable for others but did not see themselves as users.

Based upon qualitative data gathered in Step 3, the team created a final copy. The toolkit was disseminated in hard copy and in digital form (http://www.wichita.edu/aging/) to all community collaborators through the Harvey County Department on Aging. It was additionally disseminated online through the Kansas Trauma Registry, the Kansas Department of Health and Environment, and the Wichita State University Regional Institute on Aging.

## 5. Discussion

### 5.1. Unexpected Findings

Many older adult Step 3 participants felt that falls were an important concern for their aging family members and friends. However, none recognized themselves as the priority user: “Falls happen to other people, but not to me,” or “I have fallen before but my fall occurred because the floor was wet.” Initial images selected for the toolkit were of adults in the 65 to 85 year age range. Again, participants did not identify with these images, and felt images of younger, more attractive, and active older adults should be used. The participants also did not identify with the term “older adult” nor were they able to reach consensus on a more acceptable term, (e.g., elderly, senior, or aged). In an attempt to personalize the toolkit, pronouns and a specific age designation (≥65 years) were used as much as possible rather than the term “older adult.” To address the notion of “This toolkit is not for me because my fall was caused by…,” the toolkit opens with case scenarios of falls that might be attributed to non-age-related circumstances.

### 5.2. CBPR-Informed Evidence-Based Toolkit Feasible for Rural/Frontier Communities

The Falling LinKS Toolkit was designed to be sustained with limited resources and to be implemented by older adults with little to no assistance from trained professionals. Contrary to a typical top-down or expert-driven approach, a CBPR approach helps ensure practicality, community empowerment, and buy-in [14, 17]. 

The Toolkit contains an introduction with four realistic fall scenarios (i.e., tripping over a dog and slipping on wet grass); a how-to-guide for using the Toolkit; a section on personal concerns and barriers to change (i.e., I do not have time) and strategies for overcoming the barriers (i.e., make small changes on a regular basis); a questionnaire for assessing one's risk of falls; four fall risk reduction content sections (increase physical activity, review and use medications safely, identify and screen vision problems and increase home safety); a table for creating a personal fall risk reduction plan; information on the research team and acknowledgements; and references. Interspersed text boxes provide illustrations and facts about falls (i.e., as you get older, your risk of falling increases). The Toolkit presents the following four fall risk reduction content sections. 

#### 5.2.1. Physical Activity

The benefits of regular physical activity and balance training for older adults have been extensively reported, both when used alone and as a component of a multifactorial fall prevention program [18–20]. Because geriatric-specific balance and strength training programs, group exercise facilities, physical therapists, and exercise physiologists are not available for many older Kansans, the First Step to Active Health (FSAH) was chosen to serve as the basis for the physical activity module. FSAH is a flexible, multicomponent program that uses a step-by-step progression to move through four levels of fitness. It contains specific tools to assist individuals with behavior change including individual goal setting, social support, and active choices [19].

#### 5.2.2. Medications

Medication review is a valuable part of a multifactorial fall prevention program [20]. Use of high-risk medications and polypharmacy (taking more than four medications daily) are risk factors for falls, and falling is one of the most common drug side effects [21–23]. Community-dwelling older adults take an average of 6.5 medications daily and 26 different medications annually [22]. A medication review reduces fall risk by reducing the use of high-risk drugs, polypharmacy, and avoiding drug interactions and excessive dosages. Individualized reviews are more effective than general education, but individual reviews require pharmacists and prescribers who may not be available in rural counties [20]. Therefore, the medication module includes adapted versions of the American Society of Health-System Pharmacist's “My Medicine List” and discussion of the File of Life and Vial of Life tools to document medication and health information. The module includes information on safer medication use, storage, and disposal and focuses on empowerment and education to prompt older adults to seek a review from their healthcare provider when they do obtain access.

#### 5.2.3. Vision

Evidence supporting routine vision screening and correction is not as strong as the other interventions, but people with visual impairments are two to three times more likely to fall [21, 23]. Vision problems increase fall risk by decreasing the ability to detect obstacles and increasing instability. Loss of depth perception and contrast make it difficult to see or detect the height of steps, curbs, or furniture. The rates of low vision and blindness significantly increase with age, particularly for those over 65 years [24]. Optometrists and low-vision specialists are scarce in rural and frontier Kansas. Therefore, the vision module includes two effective, commonly used self-screening tools, the Amsler Grid and Visual Function Questionnaire [25]. The module offers information on coping with low vision, lighting tips, contrast, magnification, and modifications to activities of daily living to improve mobility and safety as well as resources for further vision-related assistance.

#### 5.2.4. Home Safety

The benefits of home safety evaluation and modification have been extensively reported both when used alone and as part of a multifactorial program [21]. A home safety assessment evaluates the inside and outside living environments, noting areas that may create problems or dangers, and determines appropriate safety modifications. Potentially serious environmental hazards are widespread in the homes of older adults and are particularly common in the homes of frail older adults [26]. Home safety assessments by physical or occupational therapists are not often feasible in rural settings because of the scarcity of providers. Therefore, this module includes two adapted self-assessment tools, the CDC's *Check for Safety: a Home Fall Prevention Checklist for Older Adults *and the Washington State Department of Health's *Stay Alive and Independent for Life: An Informative Guide for Adults 65+* [27, 28]. The module discusses awareness, home modification tips, and resources for assistance in completing the necessary modifications.

### 5.3. Study Limitations

Qualitative data were obtained from persons who were interested in falls and agreed to participate rather than from a systematic sampling strategy. Because of study limitations, estimates of the numbers of participants likely to reach saturation were made and recruitment was based on those estimates. But, it is unknown how closely the views expressed by the participants (*N* = 104 total) represent the larger community or if systematic sampling for research participants would have yielded different perspectives. Some segments of the older adult population of the rural county studied, specifically subgroups of a dominant religious community, were not reached. Also, qualitative data were gathered from individuals in a rural county located 30 minutes from a metropolitan area. It is not known if the perspectives and recommendations of these participants reflect those of older adults and service providers in other more distant rural counties or in frontier counties.

### 5.4. Areas for Future Research/Development

Future research should involve gaining perspectives from a broader selection of participants representing other rural counties and specifically frontier counties. The next step of this project is to evaluate older adults' perceptions and interactions with the Toolkit regarding its readability, usability, level of satisfaction, and likelihood of implementation through a pre- and postintervention assessment of knowledge and through usability testing. Ultimately, the toolkit will be tested for its ability to actually reduce fall risk.

## 6. Conclusion

The Falling LinKS Toolkit was designed for use by older adults, their families, or community advocates in rural and healthcare professional shortage areas and offers an informational and educational fall risk reduction tool. Older adults can use the toolkit to take action, individually and at home, to reduce the risk of this serious and common later life event. With the rapidly expanding older population and with significant numbers of older adults living in rural and frontier areas nationwide, self-care tools such as the Falling LinKS Toolkit will become increasingly important to the maintenance of their independence.

## Figures and Tables

**Table 1:** CBPR process for Falling LinKS Toolkit development.

Step 1		Step 2		Step 3	
Needs assessment of health providers and community volunteers	Toolkit development	Needs assessment of older adults	Toolkit development	Toolkit assessment by health providers, community volunteers, and older adults	Toolkit development
(i) Interviews (*n* = 16) (ii) Communication with representatives from Harvey County Department on Aging (iii) Focus group (*n* = 11) *Objectives: * Identify… (i) local interest (ii) need (iii) collaborators (iv) infrastructure (v) toolkit preferences for development, dissemination, and implementation *Findings: * (i) Support/interest is high (ii) Collaborators identified (iii) Resources are scarce (iv) Toolkit preferences identified	*Process Outcome: * (i) Content areas identified and researched: (1) Physical activity (2) Medications (3) Vision (4) Home safety (ii) Peer-reviewed or federal agency instruments selected, adapted, and organized (iii) Draft toolkit created	(i) Interviews (*n* = 16) (ii) Focus group (*n* = 19) *Objectives: * Identify… (i) local interest (ii) need (iii) infrastructure (iv) toolkit preferences for development, dissemination, and implementation (v) perspectives on falls and fall prevention (vi) naming strategies for the toolkit *Findings: * (i) Support/interest is high (ii) Resources are scarce (iii) Toolkit preferences identified	*Process Outcome: * (i) Instruments modified further (ii) 5th grade reading level targeted (iii) Attention to packaging, color, and readability (iv) Toolkit modified further	(i) 2 formative focus groups with health providers and community volunteers (ii) 3 formative focus groups with older adults (iii) 1 summative focus group with service and health care providers (*n* = 42) *Objectives: * Evaluate… (i) materials (ii) interest (iii) readability (iv) usability *Findings: * (i) Issues with eye appeal, interest, readability, and usability were identified	*Process Outcome: * (i) Instruments further modified (ii) Toolkit images enhanced (iii) Text condensed throughout (iv) Language changed to 1st person (v) Final toolkit created (vi) Toolkit disseminated to community partners (vii) Toolkit ready for pilot testing

Interviews: key informant interviews (individual interviews with people knowledgeable about the topic of interest). Focus Groups: semistructured interviews of a group of participants guided by a facilitator to address topics of interest.
